# A Statistical and Optimization Study on the Influence of Different Abrasive Types on Kerf Quality and Productivity during Abrasive Waterjet (AWJ) Milling of Ti-4Al-6V

**DOI:** 10.3390/ma17010011

**Published:** 2023-12-19

**Authors:** Nikolaos E. Karkalos, Lisa Dekster, Rafał Kudelski, Panagiotis Karmiris-Obratański

**Affiliations:** 1 Laboratory of Manufacturing Technology, School of Mechanical Engineering, National Technical University of Athens, 157 80 Athens, Greece; nkark@mail.ntua.gr; 2 Department of Manufacturing Systems, Faculty of Mechanical Engineering and Robotics, AGH University of Krakow, 30-059 Krakow, Poland; dekster@agh.edu.pl (L.D.); kudelski@agh.edu.pl (R.K.)

**Keywords:** abrasive waterjet milling, slot milling, garnet, silicon carbide, grey relational analysis

## Abstract

Non-conventional machining processes offer significant advantages over conventional ones, especially in terms of the productivity, cost, and surface integrity of the produced parts due to their higher flexibility. Abrasive waterjet machining, in particular, constitutes an ecologically friendly process with a negligible thermal impact on a workpiece, and it has considerable capabilities for obtaining the desired outcome by regulating some of its numerous parameters. Among these parameters, the abrasive type is particularly important due to its hardness, mesh size, and shape, which lead to considerable deviations on the obtained depth, kerf characteristics, and productivity. Thus, in this work, a comprehensive comparison is conducted on the use of garnet and silicon carbide particles for the slot milling of the Ti-6Al-4V alloy under different conditions. The capabilities of both abrasive materials are evaluated by statistical analysis regarding the depth of penetration, kerf width, kerf taper angle, and material removal rate (MRR), which are obtained under the same process conditions. Finally, a multi-objective optimization based on grey relational analysis (GRA) is performed for several different practical cases. It was found that, although silicon carbide is more efficient in optimizing individual process outputs, the use of a garnet abrasive can lead to considerably better trade-offs between two or more objectives of the machining process.

## 1. Introduction

Titanium alloys exhibit outstanding corrosion resistance, high specific strength, and low weight, making them highly advantageous in various industries, including aerospace, defense, automotive, and bio-medical [1,2,3]. However, these alloys are categorized as hard-to-cut materials due to specific inherent characteristics, including unfavorable thermal properties such as low thermal conductivity, as well as high strength and chemical reactivity [4]. In fact, previous investigations have directly underlined that machining titanium alloys presents a considerable challenge, primarily due to their poor thermal conductivity and pronounced chemical reactivity at elevated cutting temperatures [5,6]. These factors severely restrict the machinability of titanium alloys when using conventional machining techniques, thus necessitating the exploration of alternative approaches such as laser machining, electro discharge machining (EDM), and abrasive waterjet (AWJ) cutting, which have gained increasing popularity [7,8]. However, achieving high efficiency and productivity while minimizing costs and power consumption requires the careful selection of process conditions [9] that are based on both empirical knowledge and a comprehensive understanding of the underlying physics [10].

The implementation of AWJ technology has gained significant traction in the manufacturing industries for the machining of a diverse spectrum of materials encompassing both metallic and non-metallic substrates [11]. The selection of AWJ as a preferred machining process is justified by several key factors. Firstly, AWJ operates in the absence of a heat-affected zone [12], thereby ensuring minimal thermal effects on the workpiece. It is higher than other methods in terms of the material removable rate (MRR) [13], and it has better surface quality [14]. In abrasive waterjet (AWJ) machining, the process of material removal is primarily attributed to two predominant modes: cutting and the deformation/ploughing deformation erosive wear mechanism [15]. The cutting mode involves the micro-cutting of the material by the high-velocity abrasive particles present in the waterjet. These particles effectively sever the material, resulting in its removal [16]. Sharp-edged, angular particles primarily contribute to cutting deformation, while spherical abrasive particles play a significant role in ploughing deformation. Ductile erosion, on the other hand, occurs as a result of a combination of cutting wear and deformation wear mechanisms [17]. Brittle erosion occurs due to the impact of abrasive particles, which results in contact stresses and leads to crack propagation and MRR [18].

Due to the significance of accurately managing process parameters, several pertinent studies have been carried out. Alberdi and colleagues [19] made predictions about kerf geometry by examining the process parameters during the AWJM of an AA 7075-T651, and they found the stand-off distance to be the most influential factor affecting kerf width. Rabani et al. [20] applied partial non-linear differential equations for managing AWJM parameters and forecasting slots while processing a Ti6Al4V titanium alloy. The research evidenced a significant reduction in errors and a 50% enhancement in precision. This algorithmic approach allowed for the experiment setup time to be decreased by at least 200%. Using an artificial neural network, Panchal and Hafiz Shaikhb [21] performed an optimization study of AWJ parameters on specific cutting energies. The results of the AMFR revealed that the specific cutting energy was mostly affected by the jet pressure (P) and the abrasive mass flow rate. Uhlmann et al. [22] scrutinized the AWJ milling of near-net-shape fabrications of TNM-B1 titanium aluminide. The research was centered on amplifying the producible geometries by modulating the kerf width and depth, thereby augmenting the efficacy of control depth cutting operations. In a similar vein, Yuan et al. [23] utilized AWJ milling techniques to manufacture circular pockets in a titanium grade 5 alloy. Employing a Box–Behnken statistical design, they endeavored to ascertain the optimal amalgamation of machining parameters (h, ma, vt, and p). The experimental outcomes were successfully corroborated by a predictive model, registering a maximal deviation of 3.5% in the average milling depth. Moreover, in the case of pocket milling by creating overlapped slots, it was proven essential to include a lateral feed as an additional parameter when desiring to choose the most appropriate milling strategy [24].

Beyond the operational parameters, the physical properties of the abrasive particles crucially determine the outcomes in AWJM. Empirical studies indicate that abrasive particles with reduced velocities often lead to the embedment of grit within the subject material [25,26]. Concurrent research has posited that these embedded entities are, in fact, fragments of the original abrasive particulate [27,28,29,30]. The seminal work by Stachowiak and Stachowiak [31] unveiled that the morphology of the abrasive material, in superseding its hardness, largely influences grit embedment. They deduced that particles with an angular form can induce a particle embedment that is quadruple the rate of spherical-shaped glass bead particles. Subsequent investigations by Fowler et al. [32,33] delved into the AWJM treatment of titanium grade 5 with an emphasis on the implications of particle hardness and the degree of grit embedment. Their research underscored a strong correlation between the material removal rate (MRR) and the hardness and size of the particles. Furthermore, the traversal speed (vt) emerged as a pivotal process parameter. Enhancements in both vt and MRR contribute to diminished surface wave characteristics and particle embedment. In a separate study, Perec [34] employed three disparate abrasives (crushed glass, garnet, and olivine) to scrutinize the AWJ milling of a Titanium Ti6Al4V workpiece. This analytical examination encompassed the evaluation of the cutting depth capabilities of each abrasive material and the wear dynamics of the focusing tube. The results revealed that the maximum cutting depth was best attainable with garnet, followed by olivine. Contrarily, the olivine abrasive invoked the most substantial wear on the focusing tube. In subsequent studies, Perec et al. [35] and Perec [36] also compared the performance of different types of materials—with the former study being relevant to the comparison of three different abrasive materials, namely monocrystalline corundum, fused aluminum oxide (alumina-zirconia), and white fused aluminum oxide with garnet—regarding their impact on focusing tube wear, whereby the latter was relevant to the investigation of the disintegration intensity of alluvial and recycled garnet, as well as corundum and olivine. In the first study, it was shown that—although the higher hardness of corundum-based abrasives leads to an easier cutting of very hard materials—it also has a detrimental effect on focusing tube wear, resulting in an 8–16 times larger wear [35]. Moreover, in the second study, it was revealed that garnet abrasives had a greater recycling potential of up to 61% when compared to 46% for olivine and 40% for corundum [36].

Palaniyappan et al. [37] conducted a study comparing two different abrasive materials and concluded that the recycled electric waste, when used as abrasive material, exhibited similar friability and performance with commercially available garnet abrasive, but the cost was nearly half of the cost for the garnet abrasive. Khan and Haque [38] conducted a comprehensive comparison of various abrasive materials such as garnet, aluminum oxide, and silicon carbide. It was found that garnet abrasives lead to a wider taper than aluminum oxide and silicon carbide, whereas the use of silicon carbide particles leads to higher depth than aluminum oxide and garnet. Subarinthan et al. [39] compared the efficiency of recycled alumina grains to that of common garnet. Recycled alumina exhibited a higher material removal rate but also higher kerf width and surface roughness. Srinivas and Ramesh Babu [40] conducted experiments on the machining of various metal matrix composites with garnet and silicon carbide abrasives. They observed similar trends with both abrasive materials, but it was determined that silicon carbide had superior penetration ability due to its higher hardness and different geometry. Thamizhvalavan et al. [41] performed experiments on hybrid metal matrix composites by using abrasive aluminum oxide particles of three different mesh sizes (60–100) and garnet abrasive. The results showed that the use of 80-mesh aluminum oxide abrasive resulted in a higher material removal rate (MRR),as well as improved surface quality, compared to other aluminum oxide abrasives that were studied in another empirical case study. Regarding the mixing of different abrasive materials, Yu et al. [42] considered the machining parameters previously discussed alongside certain abrasive materials (garnet, alumina, and silicon carbide) and their respective mixtures. Their findings indicated that an amalgamation of 75% alumina and 25% garnet yields profound cuttings with minimal surface roughness during the AWJ machining of an aluminum alloy block. Balaji et al. [43] performed abrasive waterjet drilling on stainless steel by using mixtures of different abrasive materials, including silicon carbide, garnet, and aluminum oxide. The better-performing mixtures, regarding various output quantities and target materials, were found to be 40% garnet with 60% alumina and 60% garnet with 40% SiC. Cosansu and Cogun [44] compared to the performance of colemanite powder when used with garnet abrasive. Their findings revealed the capabilities of colemanite powder to act as an alternative to garnet, especially in terms of cost, despite its inferior hardness and overall performance (even when in a mixture with garnet). Zhu et al. [45] proposed the use of polymer abrasives for polishing purposes, and they showed that they could obtain more uniform surfaces with higher quality without the embedment phenomenon, whereas when polymer particles were mixed with particles from a moderately hard material such as silica, the embedment of particles became obvious. Thus, in that case, subsequent techniques for improving surface integrity, such as burnishing [46], would not be necessary to be employed.

As evidenced by the literature survey, although several authors have performed comparisons between different types of abrasives regarding various objectives, there appears to be a lack of extensive research on thorough analyses and comparisons of the effect of garnet and silicon carbide abrasives on various indicators, such as depth of penetration, kerf top width, kerf taper angle, as well as material removal rate and an optimization study of various process parameters in a variety of practical cases. Thus, the present study investigates the influence of abrasives on depth penetration, as well as on the kerf width, kerf taper angle, and the material removal rate during the AWJ slot milling of a titanium Ti 6Al4V alloy. Apart from an analysis on the experimental findings when using appropriate statistical methods, an optimization study based on the grey relational analysis method was also conducted to determine the optimum parameter values in different multi-objective practical cases.

## 2. Materials and Methods

In the current study, AWJ machining experiments were conducted on a Ti-6Al-4V titanium alloy workpiece. The goal was to create non-through, straight grooves using various process conditions with two different abrasives and to analyze their effect on various process outputs. More specifically, in this study, 36 experiments were conducted using three different levels of traverse speed rate (v_f_), abrasive mass flow rate (m_a_), and stand-off distance (h), as well as two levels of jet pressure (P). The experiments were separated into two groups. The first group utilized garnet as an abrasive, while the remaining group used silicon carbide. In order to design an experiment, the Taguchi orthogonal array was utilized to establish the values of the process conditions regarding v_f_, m_a_, and h, whereas the same experiments were repeated for two different jet pressure values and two different abrasive materials. The values for the selected parameters, as shown in Table 1, had a relatively wide range. This means that there can be notable variations in the results of each case while still staying within the equipment limits.

**Figure 1:**
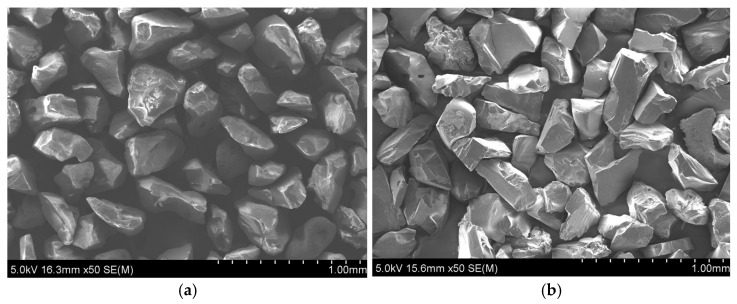
SEM micrographs of the abrasive particles: (**a**) garnet particles and (**b**) silicon carbide particles of a 60-mesh size.

All experiments were conducted on an H.G. RIDDER–Automatisierungs GmbH model HWE-1520 machine (H.G. RIDDER–Automatisierungs GmbH, Hamm, Germany). As for the experiments, the main focus of the study was to examine the influence of the abrasive type on machining characteristics in order to eliminate the influence of the size of the particles for both silicon carbide and garnet of a 60-mesh size, as shown in Figure 1. Both abrasives had an irregular geometry with sharp angles, but the silicon carbide particles seemed to be considerably rougher, a fact that was expected to be reflected in the results. The jet impingement angle was 90 deg. in every case, the diameter of the focusing tube was 1 mm, and the waterjet nozzle diameter was 0.3 mm. The workpiece dimensions were 200 mm × 35 mm × 22 mm, and the slots on the titanium workpiece were 35 mm in length.

**Figure 2:**
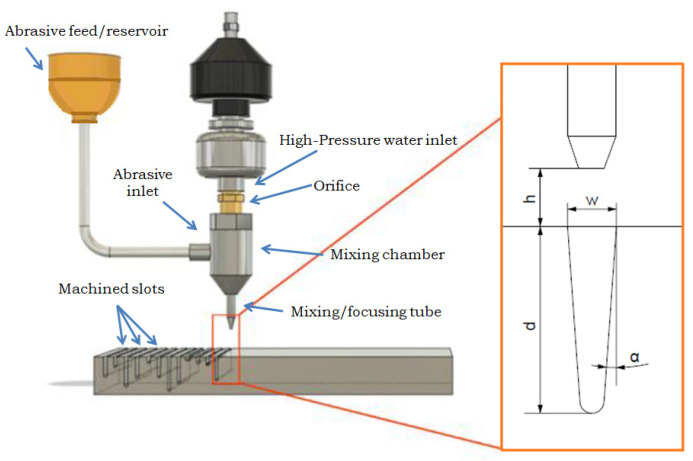
A schematic of the AWJ milling setup showing the basic parts of the experimental setup and basic geometric characteristics.

For the measurement of the dimensions of the slots, the VHX-7000 ultra-deep-field microscope (KEYENCE, Mechelen, Belgium) was used, which is a focus variation microscope (FVM) with lenses of 20 to 2000× magnification. Thus, this microscope equipped with a high-resolution camera was used to measure the depth of cut (d), top kerf width (w_t_), and kerf taper angle (α) after conducting experiments. The measurements were conducted by processing the obtained images through ImageJ software, version 1.54d. For the measurements, multiple images were acquired with a magnification of 100×, and they were processed by image stitching algorithms in order to obtain the full geometry of the grooves. In order to minimize the likelihood of measurement errors, we took three measurements of the groove depth and six measurements of the width. The statistical analysis used the arithmetic mean of the measured values. Figure 2 shows the measurement scheme for the experiments performed.

Using the Taguchi method provides a simple and effective approach for developing the optimal design of experiments to assess performance and quality. In the first stage of the analysis of the results, the Q-Dixon test was used to verify that none of the results were subject to coarse error, and none of the measured values were rejected from the dataset. To analyze the relationships between variables and to determine if there were significant differences among the groups or treatments, the ANOVA (analysis of variance) method was used. Via calculating an F-test, ANOVA assesses the significance of observed differences, thereby providing a quantitative measure of the variability between groups and enabling one to draw conclusions about relationships.

### Grey Relational Analysis (GRA) Method

To perform multi-objective optimization based on the various output quantities of the experiments, the GRA method was employed due to its robustness and relevantly simple application for various types of problems. It is important to mention that the use of optimization methods is essential in many cases, not only regarding the improvement of processes, but also in the field of inverse analysis for the identification of parameters that cannot be directly measured, e.g., material model parameters [47,48,49].

This method is relevant to grey system theory, which can handle incomplete information in order to determine the correlation between two sequences, even in situations where the amount of available data is relatively low(such as in machining experiments). This method was implemented in several steps, beginning from the initial treatment of the data up to the determination of the total relational grade for each different combination of input data, after which these relational grades were ranked to find the optimum combination [50,51].

More specifically, the first step of the implementation of GRA involves the normalization of the responses in the range between 0 and 1 in order to avoid the magnitude of the response having a considerable impact on the results (which would undermine the relative importance of other responses). Normalization was performed based on the type of each objective, e.g., whether it should be minimized, maximized, or whether it should be equal to a specific, nominal value [50,51]. In the case of minimization, the “smaller-is-better” expression can be used as follows:

(1) $$z_{ij}=\frac{\max\;y_{ij}-y_{ij}}{\max\;y_{ij}-\min\;y_{ij}}$$

where *y_ij_* represents the response for the *i*-th experimental case and the *j*-th process indicator (e.g., depth of penetration), and *z_ij_* is the normalized value. In the case of maximization, the following expression, termed as “larger-is-better”, can be employed:

(2) $$z_{ij}=\frac{y_{ij}-\min\;y_{ij}}{\max\;y_{ij}-\min\;y_{ij}}$$

Finally, when a specific, nominal value should be obtained, the “nominal-the-best” expression is used:

(3) $$z_{ij}=1-\frac{\left|y_{ij}-y_{oj}\right|}{\max(\max\;y_{ij}-y_{oj},y_{oj}-\min\;y_{ij})}$$

where *y_oj_* represents the specific value that should be obtained regarding the *j*-th indicator. Then, the second step of the GRA method was related to the calculation of the grey relational coefficient (GRC)for each case and indicator. The GRC represents a relation between the ideal value of each response and the experimentally obtained ones. The calculation of the GRC is performed based on the following formula [50,51]:

(4) $$\gamma (Z_{0},Z_{ij})=\frac{\Delta \min+\xi \Delta \max}{\Delta_{oj}(k)+\xi \Delta \max}$$

where Z_0_(*k*) represents the reference sequence(with *k* = 1, …, m and m being equal to the number of process indicators), Δ*_oj_*(*k*) represents the deviation sequence for the respective Z_0_(*k*), and Z*_ij_*(*k*) represents the comparability sequence (with Δ*_oj_*(*k*) being equal to |Z_0_(*k*) − Z*_ij_*(*k*)|, and Δmax and Δmin being the highest and lowest values of Δ*_oj_*(*k*), respectively). The distinguishing coefficient ξ can have values in the range between 0 and 1, but, in this work, it was assumed to be 0.5 as in various other studies.

After the values of the GRC were calculated, the final step of the GRA involved the calculation of the grey relational grade (GRG), which was based on the GRC values and weight factor *ω_k_*. The GRG indicates the degree of correlation between two sequences, where a higher value indicates a greater degree of correlation. The expression for calculating the GRG was the following [50,51]:

(5) $$GRG(Z_{0},Z_{ij})=\sum_{k=1}^{n}\omega_{k}\gamma (Z_{0},Z_{ij})$$

Usually, the values of *ω_k_* are considered equal for every GRC value that is related to different process indicators, but, in some cases, special methods that are related to decision making are employed in order to accurately determine the appropriate *ω_k_* values.

## 3. Results and Discussion

### 3.1. Experimental Results and Microscope Observation of the Produced Slots

After the experiments were conducted, the depth of penetration, top kerf width, and kerf taper angle were measured, whereas the MRR was determined based on the geometric quantities of the produced grooves and kinematic parameters, such as the respective traverse rate values. The experimental results are presented in Table 2.

**Figure 3:**
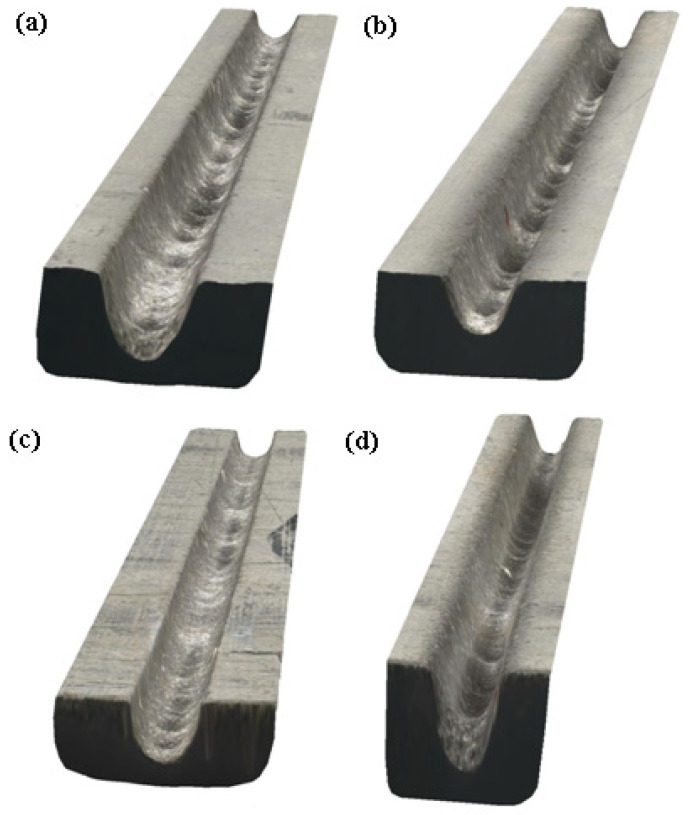
Indicative photos of the slots machined under the same conditions but with different abrasive materials (natural color): (**a**) Case no. 1 (garnet), (**b**) Case no. 5 (garnet), (**c**) Case no. 1 (SiC), and (**d**) Case no. 5 (SiC).

**Figure 4:**
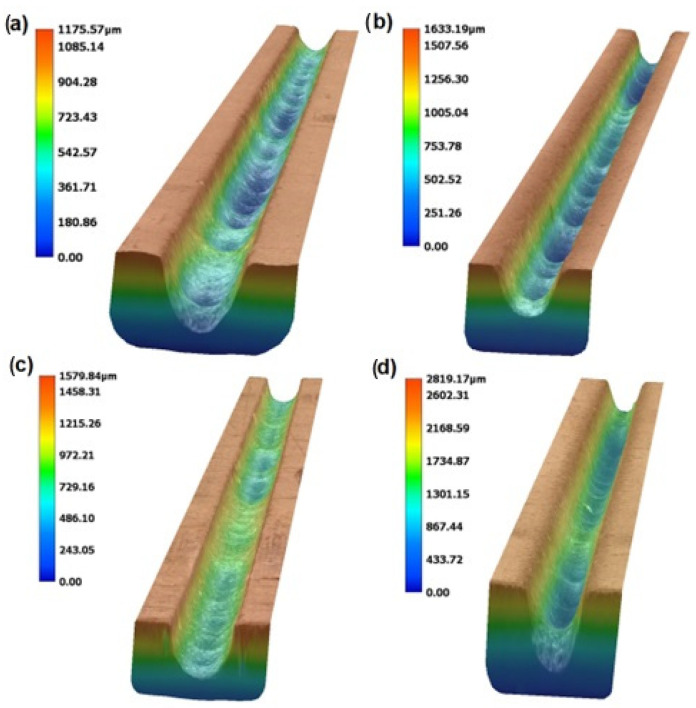
Indicative photos of the slots machined under the same conditions but with different abrasive materials (colored according to the depth of penetration): (**a**) Case no. 1 (garnet), (**b**) Case no. 5 (garnet), (**c**) Case no. 1 (SiC), and (**d**) Case no. 5 (SiC).

In Figure 3 and Figure 4, photos from two indicative slots that were machined by AWJM with both abrasive materials are depicted. From these figures and the results of Table 2, it became evident that the two different abrasives could clearly produce slots with different kerf characteristics. This is because the slots machined by silicon carbide seemed to have a larger width and a more irregular bottom surface due to the higher hardness and different geometry of silicon carbide abrasive particles, as indicated in Figure 1. These differences will be presented with more details based on the quantitative data that are shown in the following subsections.

### 3.2. Statistical Analysis of the Experimental Results

To determine the relations between the process parameters and geometric characteristics of the grooves, an analysis of the variance was performed. The graphs below were generated by the authors using the expected mean squares results. A variation analysis was carried out for the qualitative variables, such as the type of abrasive, and quantitative parameters, such as operating pressure, traverse feed rate, abrasive mass flow rate, and standoff distance. The dependent variables were the groove’s depth, width, kerf angle, and material removal rate. Setting up the diagrams in pairs allowed for comparing changes in the geometry or the influence of other parameters for garnet and silicon carbide.

The significance level for the analysis was set at 5%. The results were statistically significant and allowed for the rejection of the null hypothesis when the *p*-value was smaller than the significance level. A smaller *p*-value means that the probability of repeating the experiment with results confirming the alternative hypothesis is increased. This potentially can be useful when attempting to manufacture components with a target-specified geometry. For the part of the experiment that was conducted with silicon carbide as an abrasive, the *p*-values were generally lower. Having said that, a detailed analysis will be presented directly in this subsection.

**Figure 5:**
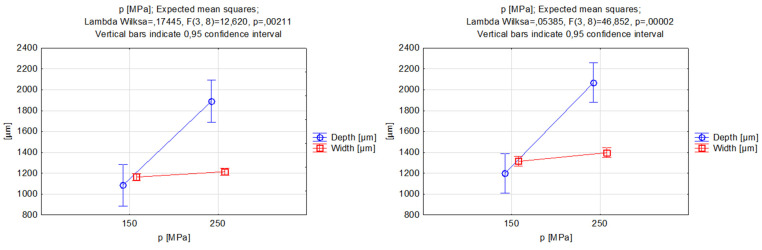
Relations between the pressure and the grooves’ depth and width. Machining with garnet (**left**) and silicon carbide (**right**).

The expected mean squares diagrams showed a relationship between the jet pressure and the groove’s width and depth for both abrasive types, as represented in Figure 5. For both the garnet and silicon carbide particles, the increasing character of the depth–pressure relationship function was clear. The depth of penetration of the AWJ in the material was reflected by an increase in the vertical cutting force. On the other hand, the relationship between the waterjet’s pressure and the width of the groove was related to a slight increase in the chosen range of values, and the difference between the values of 150 and 250 MPa was minor. Both of these conclusions were confirmed by findings from other scientific sources [52,53]. The use of harder SiC abrasive particles allowed for machining wider and deeper grooves while setting the same operating pressure. This indicated that it is possible to reduce the energy required to produce a geometry of a specified depth or width using SiC abrasive [54].The depth of the grooves after changing the abrasive material increased by 10% for the selected jet pressure range, whereas the width of the grooves increased by almost 13%.

**Figure 6:**
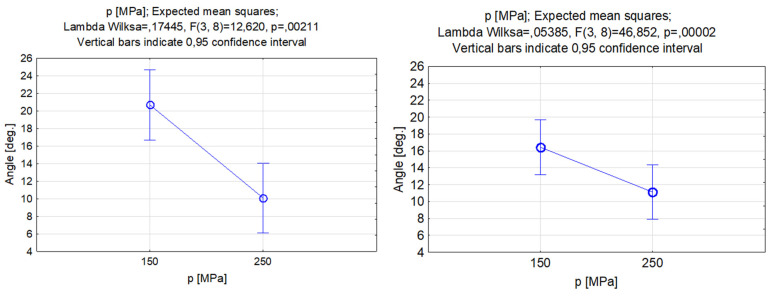
Relations between the pressure and the grooves’ kerf taper angle. Machining with garnet (**left**) and silicon carbide (**right**).

Figure 6 shows the function of the effect of the jet pressure on the kerf taper angle. Based on the analysis results, it can be deduced that increasing pressure will lead to a decrease in the inclination angle of the groove wall [55]. When using garnet as an abrasive, the expected mean squared range for angle change is larger and varies between 10 to 20 deg., whereas when using silicon carbide the kerf taper angle ranges from 11 to 16.5 deg. The results suggest that the use of silicon carbide leads to a reduction in the angle for low-pressure values. Meanwhile, a better solution to achieve the smallest possible angle for high pressures would be the use of garnet abrasive (although there was no significant difference in the extreme angle values obtained from the experiment). Pressure was not the variable that was the most significant parameter influencing the taper angle, so to decide which abrasive would be superior for a particular result, the other process parameters must also be taken into consideration.

**Figure 7:**
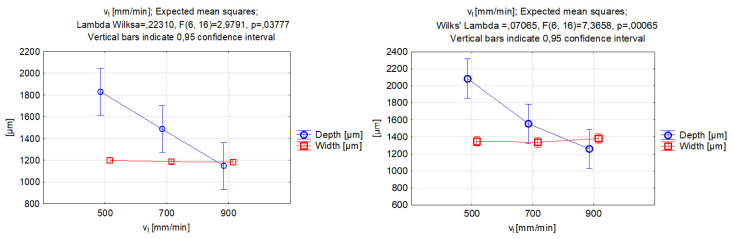
Relations between the feed rate and grooves’ depth and width. Machining with garnet (**left**) and silicon carbide (**right**).

As the experimental results in Figure 7 indicate, the lower the feed rate, the greater the depth of penetration in the workpiece, which was confirmed by the results in the literature [56]. This phenomenon was directly attributed to the difference, depending on the traverse feed rate, in the exposure time of the jet, as a lower traverse rate enables higher exposure time and thus a larger depth of penetration. The results of the analysis of variance showed that the feed rate in the studied range of the setting of this parameter, especially when silicon carbide is used, can lead to considerable variance in depth of penetration. The biggest increase in depth can be obtained using a 500 mm/min feed rate. Furthermore, the differences between the obtained depth of penetration with the two different abrasives increased for higher feed rate values. The width of the slots did not change significantly under the influence of the traverse feed rate as the width value for garnet was almost constant. Meanwhile, for silicon carbide, it gently increased for higher values of traverse feed. The width of the slots for the range of feed values from 500–900 mm/min was greater by more than 150 μm than when machining with silicon carbide. This result can be correlated with the abrasive grain disintegration phenomenon, as described in the research of Perec [57], and it might influence the abrasives differently based on their mechanical properties.

**Figure 8:**
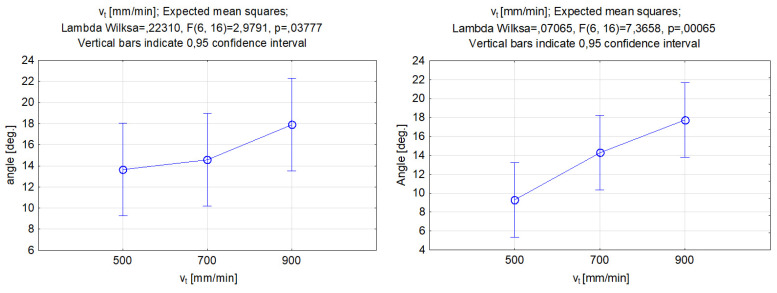
Relations between the feed rate and the grooves’ kerf taper angle. Machining with garnet (**left**) and silicon carbide (**right**).

Featured Figure 8 compares the results of the analysis of variance on the effect of the feed rate and type of abrasive on the kerf taper angle. For improving the quality of manufacturing parts made with AWJ, the most preferable case was to obtain perpendicular surfaces, so an angle closer to 0 deg. would be ideal. As is shown, the increase in feed rate increased the kerf angle in both study cases [55]. In the experiment, the smaller kerf angle values were obtained by using silicon carbide as an abrasive, and the larger variation in the kerf taper angle was obtained for this abrasive material. However, as can be seen in Figure 8, the differences between the expected mean squares for the presented feed rate values were not substantial, especially in the case of garnet abrasive, which indicated that the traverse feed rate was not so important for the regulation of the kerf taper angle when its values varied in a specific range. In the experiment, the most favorable outcome was achieved by creating a groove with a feed rate of 500 mm/min and using silicon carbide as the abrasive (based on angle reduction as the criterion).

**Figure 9:**
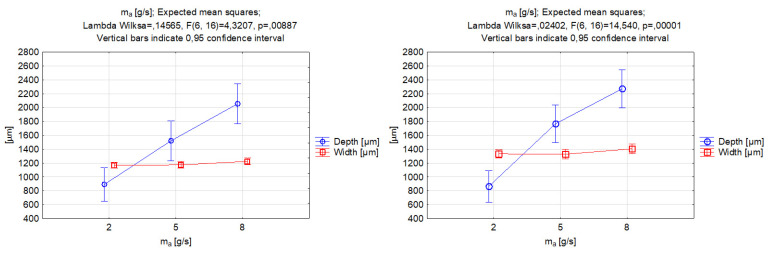
Relations between the abrasive mass flow rate and the grooves’ depth and width. Machining with garnet (**left**) and silicon carbide (**right**).

The results of the ANOVA analysis for the abrasive mass flow rate are presented in Figure 9. Upon analyzing the data, it appeared that the alterations made to the width of the grooves had no discernible impact on the function’s output value. This led us to conclude that there was no significant correlation between this particular parameter and the kerf width. However, the mechanical properties of the abrasive grains seemed to affect the obtained value of the width, which, for garnet use, varied around 1200 µm, and, for SiC particles, around 1350µm. The width values in the two cases were not significantly different for the 2 g/s value; however, increasing the abrasive mass flow caused differences in the results. The difference for the middle value was approximately 16%, and, for the highest value of the abrasive mass flow rate, the width increased by approximately 10%.

On the other hand, the abrasive mass flow rate had a significant effect on the depth of penetration, with depth values clearly increasing for higher mass flow rate values. Other studies that have been carried out have also indicated that increasing the mass flow of the abrasive will enable a deeper groove to be made due to the larger number of particles impacting the workpiece surface at the same time [58]. The difference in the minimum and maximum obtained depth with respect to abrasive mass flow rate was similar for both abrasive materials; however, there was evidence to conclude that the major difference in the material depth penetration between the two abrasive materials occurred for the middle value of the processing parameter in question. The outcome might be connected with the disintegration of the abrasive particles in a mixing chamber, as softer garnet particles can become more fractured at a larger mass flow rate, while this process is limited when increasing the number of silicon carbide particles(which are extremely hard).

**Figure 10:**
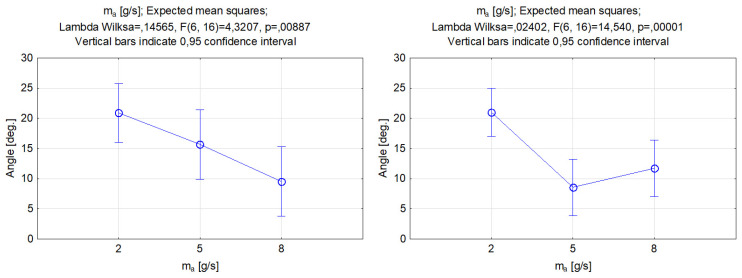
Relations between the abrasive mass flow rate and the grooves’ kerf taper angle. Machining with garnet (**left**) and silicon carbide (**right**).

The correlations between the abrasive mass flow rate and the grooves’ kerf taper angle for the examined abrasives is featured in Figure 10. Within the change from a 2 to 5 g/s abrasive mass flow rate, the value of the kerf taper angle declined. The reduction was more rapid in the process that was performed with silicon carbide abrasive. The function shape for the lowest abrasive mass flow rate in both cases did not differ significantly. However, the kerf taper angle values obtained with a parameter value of 5 g/s were quite different, namely for garnet 15.7 and silicon carbide 8.6 deg. The selected mass flow value and use of silicon carbide enabled a substantial reduction in the kerf taper angle. Increasing the abrasive mass flow rate for the SiC particles to 8 g/s indicated the deterioration of the output parameter and the increase in the kerf angle. However, the use of garnet allowed for a steady decrease in the value of the output parameter. The increase in the abrasive mass flow rate caused an increase in the energy transfer from the waterjet to the abrasive particles. Improvement in the energy transfer rate was possible due to the increase in the mixed abrasive numbers caused by collision dissipation [42]. Within the energy transfer of the hard particles of silicon carbide, the energy augmented and affected the increased material removal and deterioration of results in the kerf taper angle. A greater number of very hard silicon carbide particles are less likely to fragment compared to garnet; thus, large particles of silicon carbide cause a decrease in the coherence of the jet and detachment of the larger particles of titanium alloy, which leads to an increase in the angle of inclination of the groove wall.

**Figure 11:**
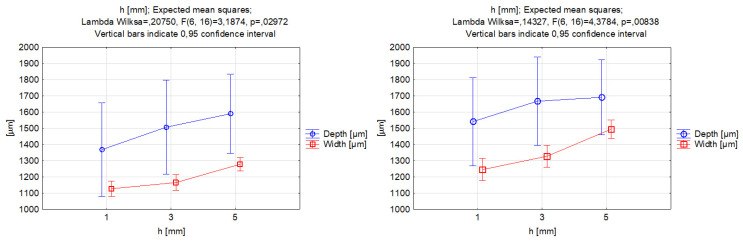
Relations between the stand-off distance and grooves’ depth and width. Machining with garnet (**left**) and silicon carbide (**right**).

It can be seen, in Figure 11, that the effects of changing the stand-off distance for both abrasive materials were similar, and this was evident by comparing the shapes of the functions. By increasing the stand-off distance, we could obtain a higher value of depth penetration in the groove, but it was evident from the data in Figure 9 that the differences between the different levels of standoff distances were not statistically significant. Thus, the stand-off distance was not a significant factor for controlling the depth of the cut. The increase might be connected to other factors such as abrasive mass flow rate, traverse feed, and high operating pressure. For the groove depth, a change of 6% in the highest value between garnet and SiC was observed. Accordingly, for the width of the groove, an increasing trend was also obtained, with more significant differences than the depth of cut. Furthermore, the highest value was achieved at a 5 mm distance between the cutting head and the workpiece, with the difference between the garnet and silicon carbide reaching 17%.

**Figure 12:**
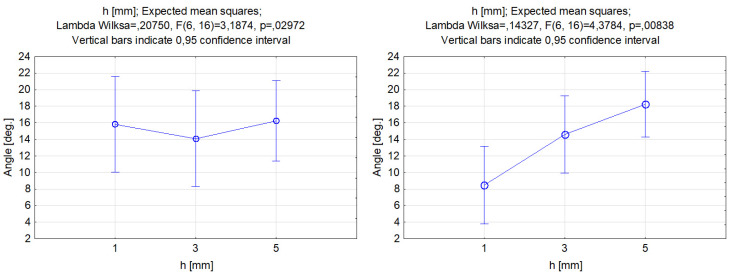
Relations between the stand-off distance and the grooves’ kerf taper angle. Machining with garnet (**left**) and silicon carbide (**right**).

The experimental data plotted in Figure 12 show the effect of the stand-off distance on the kerf taper angle. As can be seen, the use of different abrasive types influenced the trend of the kerf taper angle differently. Changing the distance of the nozzle over the material when the garnet abrasive was used had little effect on the result. The graph shows an oscillation of the kerf angle by about 2 deg., and the difference between the different levels was not statistically significant. Other scientific studies have confirmed that the stand-off distance in experiments that used garnet as an abrasive did not significantly affect the taper kerf angle [59]. The graph on the right clearly illustrates the considerably greater effect of the stand-off distance on the taper angle if silicon carbide is used. The change between the lowest and the highest values of the stand-off distance varied from approximately 8 to 18 deg. In the selected range of the input variable, an almost linear increase in the kerf angle can be found when increasing the distance of the nozzle from the sample surface. When the nozzle moved away from the material, the water jet expanded and altered the angle at which the particles hit the sample. The hardness of the silicon carbide material, among other factors, allows it to maintain its kinetic energy even if the distance or angle at which the particles hit the workpiece changes. The SiC hardness was shown to have a direct impact coherence of the jet and outcome [60]. The results of the analysis indicated, according to the results of Aydin et al. [61], that the use of silicon carbide as the abrasive particles can improve the quality of the cut in the material and reduce the kerf angle.

Furthermore, ANOVA was also used to identify the parameters with the greatest impact on the material removal rate (MRR). The MRR is an indicator of the efficiency of a machining process. A higher MRR means that more material is being removed in a time unit, thus leading to increased productivity. Moreover, the MRR is directly related to the machining speed, and higher MRR values imply faster machining rates, which can be crucial in industries where production time is critical. Finally, the MRR can provide insights into the performance of a machining operation.

**Figure 13:**
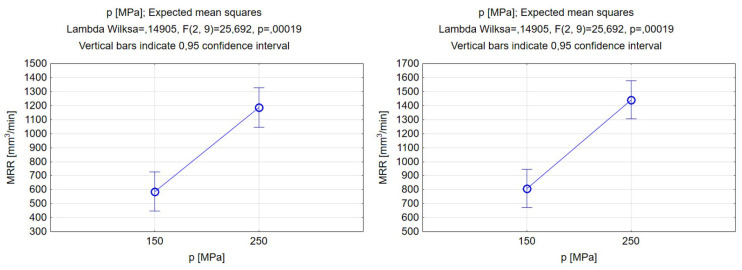
Relations between the pressure and material removal rate. Machining with garnet (**left**) and silicon carbide (**right**).

Figure 13 displays the correlation between the pressure of the jet and the material removal rate. Based on the results, it is evident that increasing the value of the jet pressure leads to a significant rise in the MRR. The increase in the MRR is attributed to the erosion and abrasion occurring at higher jet pressures during continuous machining. Higher pressure values lead to higher energy, which allows for removing more material in a time unit [62]. A subsequent conclusion can be made that the silicon carbide abrasive type allows for the removal of more material than garnet when using the same pressure value in a unit of time. The MRR values corresponding to the lower pressure for garnet and silicon carbide abrasives were 586.8 and 808.6 mm^3^/min, respectively, while those corresponding to 250 MPa were 1186.1 and 1441.4 mm^3^/min, respectively. The impact of the abrasive type was stronger for the lower pressure value as the MRR increased by more than twofold. After an increase in jet pressure, the difference between the MRR values decreased but remained significant. These findings are in line with the work of Fowler et al., where it was found that the material removal rate increases significantly as the hardness of the abrasive particles increases [32].

**Figure 14:**
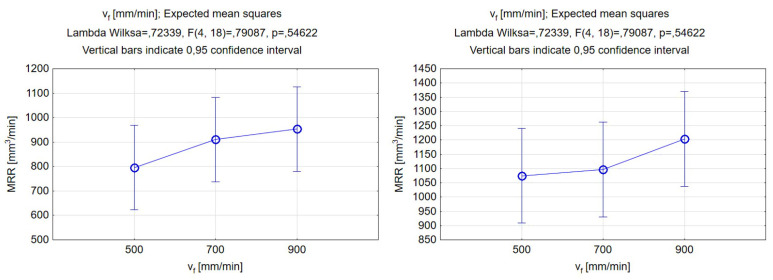
Relations between the traverse feed rate and material removal rate. Machining with garnet (**left**) and silicon carbide (**right**).

According to the findings depicted in Figure 14, there was a weak correlation between the traverse feed rate and the material removal rate. The data suggested that, as the feed rate increases, the MRR value also increases, which is a phenomenon that has already been observed in the case of hard-to-cut materials [62]. Higher feed rates allow for a quicker cut of a particular section of material. However, given that the differences between different levels of traverse feed rate are not statistically significant, it can be concluded that the main contribution to the increase in MRR is the enlargement of the incision profile of the groove for higher jet pressure and abrasive mass flow rate, rather than the increase in traverse feed. The differences in the results corresponding to a 500, 700, and 900 mm/min feed rate did not exceed 115 mm^3^/min, but major differences could be observed between the results obtained with different types of abrasives. The silicon carbide abrasive showed better material removal rate values. By switching from garnet to silicon carbide as the abrasive type, it was possible to remove an additional 250 mm^3^ per minute with a traverse feed rate of 900 mm/min.

**Figure 15:**
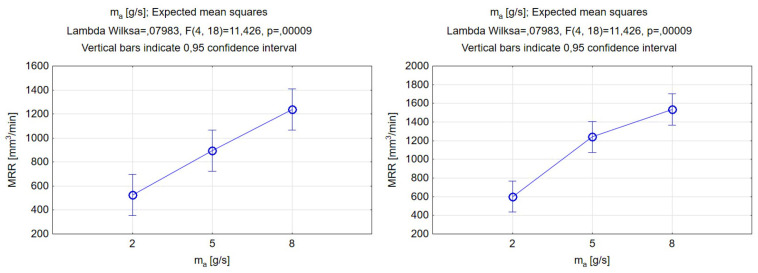
Relations between the abrasive mass flow rate and material removal rate. Machining with garnet (**left**) and silicon carbide (**right**).

As was already mentioned, the increase in abrasive mass flow rate as a process parameter refers to more particles impacting the surface of the workpiece in a unit of time. As more energy can be transferred, a larger amount of material can be removed from the sample. These properties are reflected in the results, which can be found in Figure 15. The correlation curve was upward, meaning that the higher the parameter m_a_, the greater the MRR. The difference between the lowest and the highest value of the abrasive mass flow rate for the garnet equaled 713.4, whereas, for the SiC, it was 934.2 mm^3^/min. This indicated that the abrasive mass flow rate is a very significant parameter for the MRR. Moreover, the MRR values increased by using silicon carbide relative to garnet by 74.5, 345.7, and 295.3 mm^3^/min for the respective three levels of the abrasive mass flow rate.

**Figure 16:**
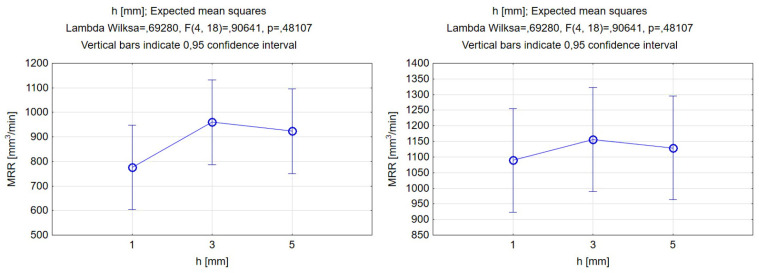
Relations between the stand-off distance and material removal rate. Machining with garnet (**left**) and silicon carbide (**right**).

The results corresponding to the relationship between the MRR and stand-off distance are presented in Figure 16. Regarding the stand-off distance, for values within the chosen range, there was a slight variation in the MRR value, with the differences being correlated with the phenomenon of waterjet expansion. This was especially the case for the silicon carbide abrasive, wherein the differences between the different levels were not significant. The slight differences can be directly explained given the flow field of the waterjet. From 1 to 3 mm, the MRR increased, then, in both cases, it started to decrease. This may be related to the fact that, up to a certain stand-off distance, the area of effect of the jet on the surface over which the material was removed increases; thus, the fluid jet was able to remove more material per unit time. However, when exceeding some of the limiting values of the stand-off distance, the expanded jet loses enough energy, and a greater angle of impact of the waterjet and abrasive on the material is not beneficial for achieving better MRR values. Functional shapes in both cases coincided with a mechanism that confirmed the tendency. As can be seen from previous considerations, silicon carbide achieves improved MRR values, and this can also be read from the differences in the diagrams above.

### 3.3. Single Objective Optimization of the Process Outputs

The Taguchi method is a statistical approach that is widely used in manufacturing for process optimization and robust design. One key aspect is calculating the ETA (estimated time average) value, which measures process quality. By optimizing the ETA, the manufacturers can achieve better process capability and product quality. This section explores the Taguchi method’s application in calculating ETA and optimizing manufacturing results. It offers advantages such as reducing variability, improving reliability, and minimizing costs.

**Figure 17:**
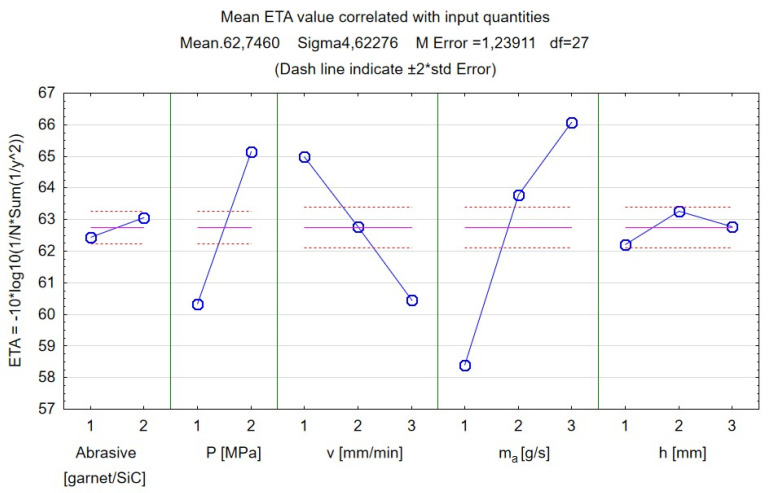
Results of the Taguchi analysis for the depth of the penetration. Signal-to-noise ratio: “Larger is better”.

In the Taguchi method, the signal-to-noise (S/N) ratio is expressed as a log transformation of the mean squared deviation, which is used as a measure to analyze the experimental results [63]. Figure 17 shows the results of the Taguchi method for the optimization of the depth of penetration. The ETA value was calculated based on the signal-to-noise ratio equation, which was created for finding the maximized response. The ETA here is the S/N ratio; n is the number of measurements for a particular quantity; and “y” is the corresponding characteristic. Each level of the input parameters was assigned an ETA value, which are represented as a circle on the graph. The higher the ETA value is, the better for achieving the aim of creating the deepest groove. The deepest groove can be made by using the following parameters: P = 250 MPa, v_t_ = 500 mm/min, m_a_ = 6 g/s, h = 3 mm, and the choice of silicon carbide as an abrasive.

**Figure 18:**
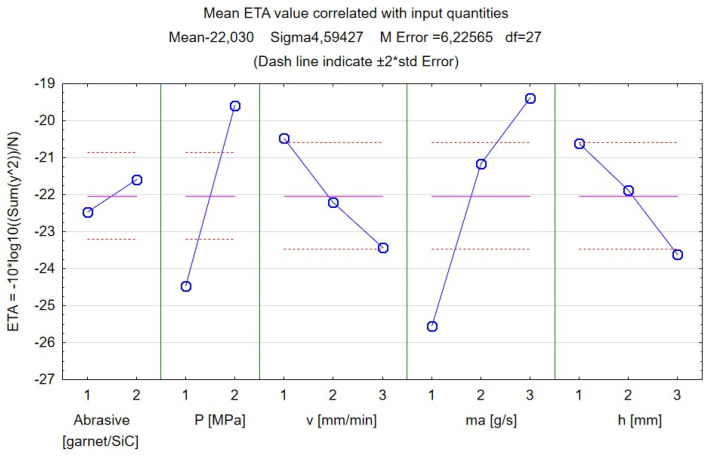
Results of the Taguchi analysis for the kerf taper angle. Signal-to-noise ratio: “Smaller is better”.

In the case of the kerf taper angle, the signal-to-noise ratio was selected in order to minimize the response. The ETA value was calculated using the formula presented in Figure 18. A multiplier of −10 ensured that the coefficient measured the inverse of an undesirable feature; in this case, this was a large inclination of the groove wall, which indicates low cutting quality. Maximizing the ETA will result in improved quality.

It is possible to reduce the kerf taper angle by adjusting the parameters appropriately. In this study case, it was deduced that—by using the parameters’ values of P = 250 MPa, v_t_ = 500 mm/min, m_a_ = 8 g/s, h = 1 mm, and SiC particles as an abrasive—we can achieve the lowest value of the kerf taper angle.

By monitoring and optimizing the MRR, manufacturers can assess the effectiveness of their machining processes and make adjustments to improve efficiency and productivity. Thus, we also utilized the Taguchi method to attain the optimal parameters that yield a high MRR value.

**Figure 19:**
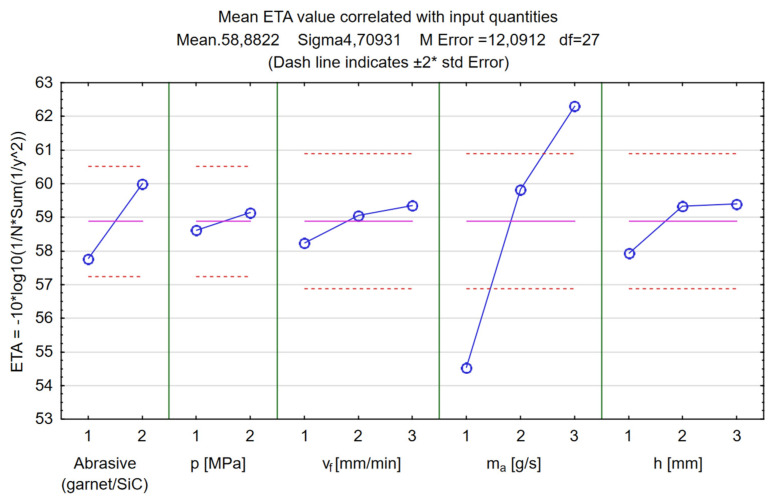
Results of the Taguchi analysis for the MRR (material removal rate). Signal-to-noise ratio: “Larger is better”.

In Figure 19, it can be seen that the Taguchi method was used to calculate the ETA value, for which the signal-to-noise ratio equation was created to find the maximized response. To achieve the highest material removal rate, it is important to maximize the response by identifying the most appropriate process parameter values. The expected ratio of S/N under optimal conditions was a value of 64.66. The highest value of the MRR can be expected when the pressure is set to 250 MPa, the traverse feed rate to 900 mm/min, the abrasive mass flow rate to 8 g/s, the stand-off distance to 5 mm, and silicon carbide is the type of abrasive.

### 3.4. Multi-Objective Optimization of Process Outputs

After the experimental results were analyzed and the correlations of the input parameters with the various outputs of the AWJM process were determined, it was important to determine the optimum parameters that not only regarded the separate outputs of the process, but also regarded multiple objectives with a more practical meaning (which involve a combination of process outputs). For that reason, three different multi-objective optimization cases were examined, and these were relevant to some of the most important objectives for the AWJM process, such as the achievement of a specific depth of cut (controlled depth milling), the minimization of kerf defects, and the maximization of productivity (as expressed through the MRR). It is worth noting that, in every case, different objectives may be contradictory so that the trade-off between them will not be a trivial problem. Optimization was based on Grey Relational Analysis, which is a rapid and robust method used to achieve the desired outcome directly based on the experimental results and Grey theory.

#### 3.4.1. First Optimization Case

The first optimization case was relevant to the achievement of controlled depth milling for the slots and, at the same time, the lowest possible kerf width. This test case can be related to a certain specification made by the customers, who desire the production of grooves with a specific depth and minimum possible deviations in the kerf width. In order to obtain more comprehensive results, this case will be further divided into two cases, one for a lower depth, namely 0.5 mm, and the second for a higher depth, namely 3 mm. As the objectives were specific depths and the minimum kerf top width, the nominal-the-best and smaller-is-better functions were employed.

**Figure 20:**
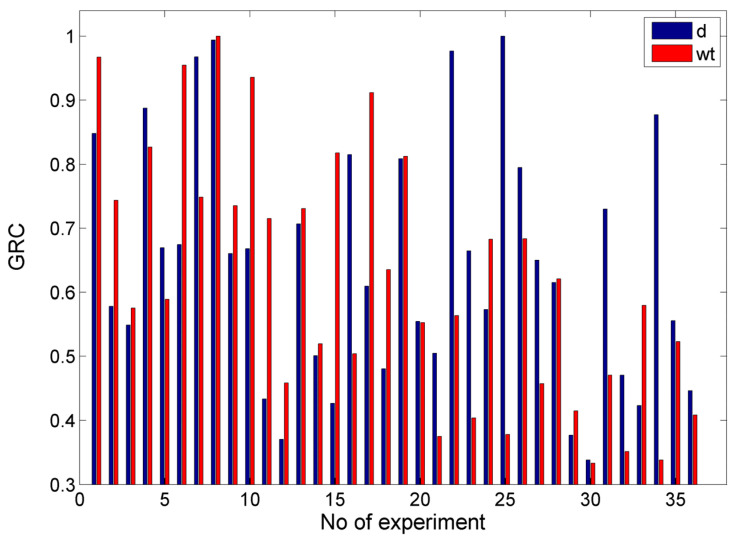
The GRC values for the case with the objectives d = 0.5 mm and min wt.

In Figure 20, the values of the GRC for the case with objectives d = 0.5 mm and min w_t_ are presented. From these results, it can be seen that the GRC values for the two objectives were high and close for the experimental cases with relatively less intense conditions, which led to both lower depths and smaller kerf widths. However, when the conditions were more intensive, e.g., under higher abrasive mass flow rates or pressure, the GRC values were smaller. After calculating the GRG and ranking the alternatives, the optimum solution was a P = 150 MPa, vt = 900 mm/min, ma = 5 g/s, h = 1 mm, and garnet as abrasive material (with which a depth of 0.532 mm and width of 1.094 mm was achieved). This result was quite good as the depth was close to the desired one and the width was the minimum width obtained experimentally. Moreover, in Table 3, the three highest ranking alternatives are presented along with their respective outputs, thereby showing that the next alternatives were clearly inferior as they resulted in higher depth and width values, whereby the depth in the third one was over 0.2 mm larger. Although the values of some of the process parameters were very different for these solutions, the pressure and abrasive material were the same as these parameters were crucial for obtaining lower depths and kerf widths.

Although the GRA, contrary to stochastic optimization methods, cannot determine the optimum solutions from a large number of random combinations within the search space, it can rapidly determine favorable solutions given that several alternatives are possible, as in the present case. The reason that it cannot reach the exact desired results for each objective is that it actually provides the best trade-off between them, given that it is not always possible in practice due to technological limitations to achieve the best value for every objective. However, this result is rather important as it can provide valuable suggestions to the machine tool operator in order to appropriately adjust the process parameters so as to achieve the best possible solution.

**Figure 21:**
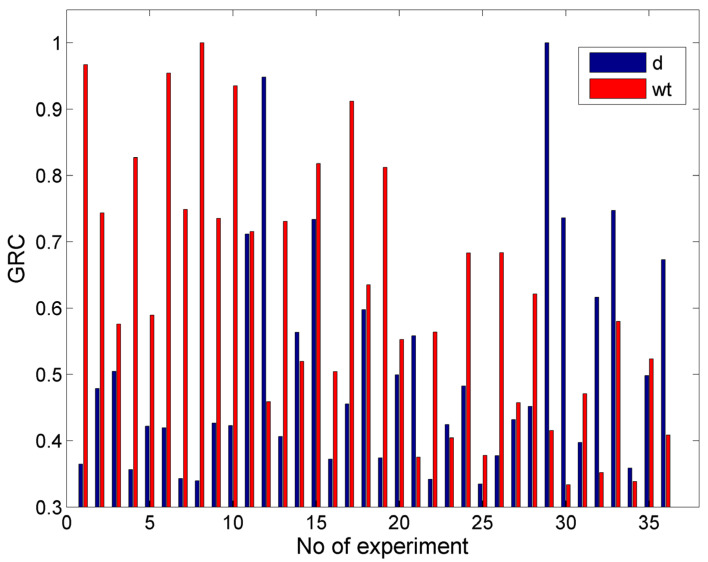
GRC values for the case with the objectives d = 3.0 mm and min wt.

In Figure 21, the values of the GRC for the case with objectives d = 3.0 mm and min w_t_ are presented. In that case, the results seemed different from the previous cases due to the different objectives for the depth. Based on the previously conducted analysis of the experimental results, it was obvious that the conditions that can lead to higher depths are not appropriate for obtaining the minimum kerf width; thus, it is more difficult to reach a favorable compromise for these targets. After calculating the GRC and performing the ranking of different alternatives, it was determined that the optimum parameter values were P = 250 MPa, vt = 700 mm/min, ma = 8 g/s, h = 1 mm, and garnet as abrasive material, with which a depth value of 2.538 mm and a kerf top width value of 1.145 mm were obtained. The depth value was almost 0.5 mm lower than the ideal depth, and the value of the width was almost 5% higher than the ideal one. Moreover, in Table 4, the three highest ranking alternatives are presented, as well as the respective outputs, thereby indicating that it was not possible to obtain a better solution as increased values of depth led to a large increase in the top kerf width and to unfavorable trade-offs. Compared to the previous case, the pressure value was again the same for the three best solutions. However, it was increased to 250 MPa and the abrasive mass flow rate was increased from 5 to 8 g/s in order to achieve a higher depth of penetration, whereas for one of the non-optimal solutions, the abrasive material was silicon carbide. This was a notable difference from the single objective optimization cases of Section 3.3, where the best option for the optimization of the individual outputs was to choose silicon carbide as the abrasive as it can lead to extreme solutions, but it was not effective for the simultaneous achievement of two or more objectives (especially when they are contradictory).

#### 3.4.2. Second Optimization Case

The second optimization case was relevant for obtaining the lowest kerf width, while, at the same time, achieving MRR values (productivity) that were the highest possible. This combination of objectives also had practical importance, as the two main goals of every manufacturing process are high quality and low machining time (or high productivity). As the objectives are the minimum kerf top width and maximum MRR, the smaller-is-better and larger-is-better functions were employed for the normalization of the results.

**Figure 22:**
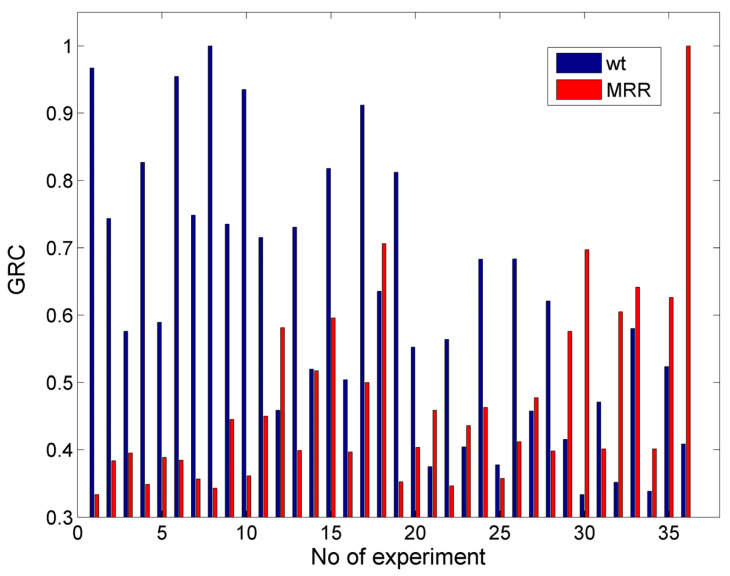
The GRC values for the case with the objectives min wt and max MRR.

In Figure 22, the values of the GRC for the case with objectives min w_t_ and max MRR are presented. From these results, it can be seen that, in most cases, the values of the GRC for the w_t_ and MRR were considerably different as the achievement of a low kerf width occurs when process conditions are less intense, which is a process that leads to a low MRR as well. After calculating the GRG and ranking the alternatives, the optimum solution was determined as a P = 250 MPa, v_t_ = 700 mm/min, m_a_ = 8 g/s, h = 1 mm, and garnet as the abrasive material, with which a top kerf width of 1.145 mm and a MRR of 1593.649 mm^3^/min can be achieved. Under these conditions, the top kerf width is almost 5% higher than the minimum one, and the MRR value is 30% less than the maximum MRR. In Table 5, the three highest ranking alternatives are presented along with their respective outputs, thus showing that the less optimal solutions lead either to a lower MRR or higher kerf width, which means a less favorable trade-off. Although the recommended pressure was the same in every case, in one solution, the preferred abrasive material was silicon carbide, which indeed increased the MRR considerably to its maximum value, but it also led to a much wider slot. It has been demonstrated that, while machining with silicon carbide can increase depth and the MRR, as well as decrease the kerf taper angle, it cannot achieve a satisfactory compromise between the two conflicting objectives.

The optimization results indicate that, in order to obtain a low kerf width and high MRR, the maximum pressure, moderate traverse speed, maximum abrasive mass flow rate, minimum standoff distance, and garnet material (less hard) should be selected. Thus, the compromise between the two objectives is mainly regulated by using the relatively intense parameters regarding pressure and abrasive mass flow rate to increase the MRR, but also a less hard abrasive in order to avoid large widths.

#### 3.4.3. The Third Optimization Case

The third optimization case was the most complex as it included three different objectives, namely the specific depth of penetration along with the minimum kerf width and maximum MRR. As in Section 3.4.1, this case was divided into two sub-cases, with two different desired depth values, namely 0.5 and 3.0 mm. As the objectives were specific depths, the minimum kerf top width, and maximum MRR, the nominal-the-best, smaller-is-better, and larger-is-better functions were employed.

**Figure 23:**
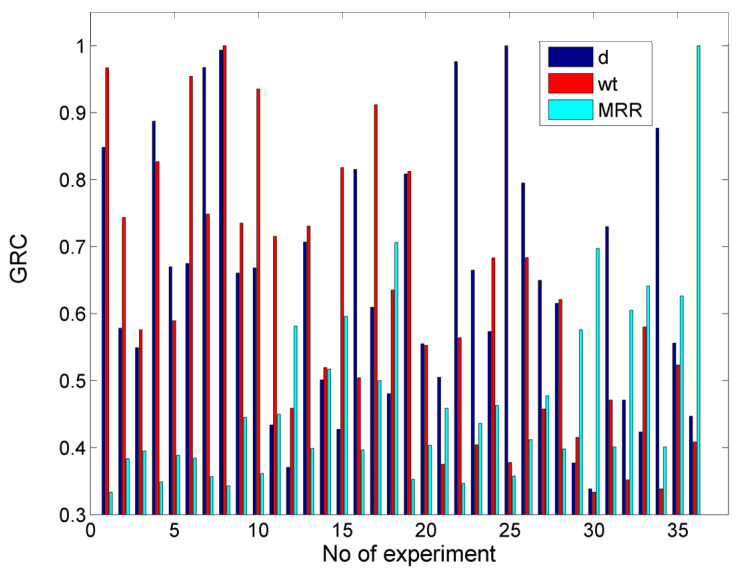
The GRC values for the case with the objectives d = 0.5, min wt and max MRR.

In Figure 23, the values of the GRC for the case with objectives d = 0.5 mm, min w_t_, and max MRR are presented. From these results, it can be seen that the GRC values for the MRR deviated considerably from the GRC values for depth and width in many cases, as obtaining a high MRR is contrary to obtaining a low depth and width. After calculating the GRG and ranking the alternatives, the optimum solution was determined as a P = 150 MPa, vt = 900 mm/min, ma = 5 g/s, h = 1 mm, and garnet as the abrasive material, whereby a depth value of 0.532 mm, a top kerf width of 1.094 mm, and a MRR of 351.845 mm^3^/min can be achieved. This solution was the same as the one determined in Section 3.4.1 for a low depth and kerf width, which reached acceptable values but were not favorable for MRR, which was close to its lowest value. These results can be justified by the fact that the achievement of the first two objectives contradicted the achievement of the third one and, given that an equal weight was used for each objective, the final solution was more beneficial for depth and width rather than the MRR.

Moreover, in Table 6, the three highest ranking alternatives are presented along with their respective outputs, thereby showing that—for different reasons—the other alternatives have considerable deviations from the optimum solution, as the second one leads to over a 0.2 mm higher depth and an even lower MRR, whereas the third one leads to both a higher depth and width (although it provides an improvement in the MRR). In every alternative case, garnet was suggested as the abrasive material as it leads to lower depth and width values. It can be generally noted that it is rather difficult to achieve a specific depth along with a better kerf quality and MRR, but at least the optimization process can provide the operator with a useful suggestion in order to choose the machining strategy.

**Figure 24:**
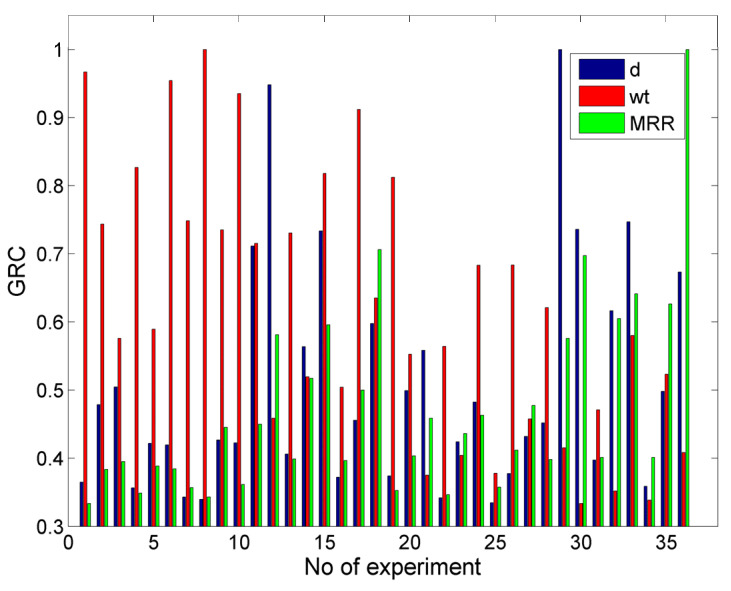
The GRC values for the case with the objectives d = 3.0 mm, min wt, and max MRR.

In Figure 24, the values ofthe GRC for the case with the objectives d = 3.0 mm, min w_t_, and max MRR are presented. From these results, it can be seen that, contrary to the previous case, it was also possible to observe high values of the GRC for depth and MRR simultaneously. This is because the achievement of a higher depth is in line with achievinga higher MRR and is opposite to the goal of achieving the lowest width.After calculating the GRG and ranking the alternatives, the optimum solution was determined as a P = 250 MPa, vt = 700 mm/min, ma = 8 g/s, h = 1 mm, and garnet as the abrasive material, with which a depth value of 2.538 mm, a top kerf width of 1.145 mm, and a MRR of 1593.649 mm^3^/min can be achieved. This result could be justified as the greater similarity between the first and third objectives led to a considerably higher result for the MRR, although the width was slightly higher than the optimum value, and the depth was almost 0.5 mm lower than the desired one.

Moreover, in Table 7, the three highest ranking alternatives are presented along with their respective outputs, thus showing that the alternatives are inacceptable due to the fact that they lead to either much higher width values or a lower MRR. Again, the recommended pressure value was the same in every case, but it was worth noting that this was the only case that the second and third best alternatives were related to the use of silicon carbide particles. This result can be attributed to the fact that a higher depth, along with a higher MRR, was desired.

Finally, it can be concluded that the use of the GRA method led to reasonable results regarding the optimum parameter values for different practical cases. Although this method has some shortcomings, the results that were still obtained through this simple, rapid, and reliable procedure are important for providing suggestions for the operators or engineers who design the required manufacturing processes for various products.

## 4. Conclusions

In this study, AWJ milling experiments were conducted to investigate the interplay between various process parameters and outcomes like the depth of penetration, kerf width, kerf taper angle, and material removal rate. The abrasive materials—garnet and silicon carbide—were employed in the machining process of Ti-4Al-6V titanium alloy. Statistical analysis via ANOVA provided insights into the significance of the process parameters. Additionally, single- and multi-objective optimization cases were explored, thereby unveiling the process capabilities across diverse practical scenarios and identifying the optimum parameters.

Based on this work, the following conclusions can be inferred:

- The use of silicon carbide abrasives enhances AWJ machining, thereby yielding greater depths and a MRR, as well as reduced kerf taper angles under similar conditions. While SiC usage reduces the machining time and energy consumption, it also leads to increased kerf width and accelerated nozzle wear.
- Single-objective optimization identified the optimal input parameters within a specified range for achieving the maximum groove depth and minimum kerf taper angle. Moreover, regarding the MRR, a significant increase can be obtained with the use of silicon carbide compared with garnet use.
- Conversely, multi-objective optimization when using the GRA method in various practical scenarios unveiled additional insights into the capabilities of the AWJM process. In controlled depth milling, with a focus on minimizing kerf width, garnet emerged as the optimal abrasive. Its usage allowed for an improved approximation of the desired depth values, and it also simultaneously achieved low kerf width values and a sufficient MRR.

In conclusion, although silicon carbide was shown to be more effective regarding depth, kerf taper angle, and the MRR in single-objective optimization studies, garnet was more efficient when multiple objectives were considered as it is related to a better capability of reduced kerf width as well.

## Figures and Tables

**Table 1:** Process parameters values (for both abrasive materials).

No.	Jet Pressure (MPa)	Traverse Feed Rate (mm/min)	Abrasive Mass Flow Rate (g/s)	Stand-Off Distance (mm)
1	150	500	2	1
2	150	500	5	3
3	150	500	8	5
4	150	700	2	3
5	150	700	5	5
6	150	700	8	1
7	150	900	2	5
8	150	900	5	1
9	150	900	8	3
10	250	500	2	1
11	250	500	5	3
12	250	500	8	5
13	250	700	2	3
14	250	700	5	5
15	250	700	8	1
16	250	900	2	5
17	250	900	5	1
18	250	900	8	3

**Table 2:** Experiment results for the AWJ milling. The results for the slots machined with garnet are presented on the left and those machined with SiC on the right.

No.	d (µm)	w_t_ (µm)	α (deg.)	MRR (mm^3^/min)	No.	d (µm)	w_t_ (µm)	α (deg.)	MRR (mm^3^/min)
1	790.810	1101.767	27.770	270.990	1	877.480	1146.492	10.220	433.603
2	1616.703	1172.378	12.240	664.185	2	1726.720	1277.933	11.810	791.605
3	1754.653	1261.408	13.430	739.078	3	1994.743	1472.832	10.770	1090.523
4	712.147	1141.668	25.300	401.314	4	558.177	1269.773	27.720	381.532
5	1262.743	1252.365	20.230	695.659	5	1278.603	1429.195	14.730	978.298
6	1245.450	1104.878	15.240	667.430	6	1639.627	1199.443	8.030	1111.164
7	572.290	1170.277	24.940	465.689	7	478.093	1468.102	38.000	470.978
8	531.897	1094.028	34.020	351.845	8	909.307	1199.140	10.620	841.814
9	1293.500	1175.890	12.870	1024.86	9	1331.270	1363.540	15.960	1177.547
10	1266.950	1109.718	14.100	501.384	10	1460.880	1232.620	7.560	758.734
11	2483.850	1184.500	7.800	1048.501	11	3003.560	1414.145	7.430	1535.497
12	3072.490	1362.112	6.540	1551.412	12	3457.523	1548.220	7.980	1838.586
13	1144.907	1177.772	11.070	764.386	13	1077.297	1349.282	16.480	777.169
14	2017.420	1303.998	10.010	1338.632	14	2208.857	1512.865	11.890	1620.090
15	2537.590	1144.638	5.570	1593.649	15	2568.543	1258.582	6.780	1713.854
16	861.990	1317.603	22.220	749.011	16	732.133	1538.195	26.020	778.048
17	1484.097	1116.005	6.320	1271.086	17	1721.937	1301.010	7.250	1676.748
18	2144.347	1224.380	6.970	1857.013	18	2382.173	1422.975	8.650	2273.834

**Table 3:** The three best ranking alternatives for the objectives d = 0.5 mm and min w_t_.

No.	P (MPa)	v_t_ (mm/min)	m_a_ (g/s)	h (mm)	Abrasive Material	d (mm)	w_t_ (mm)
1	150	900	5	1	Garnet	0.532	1.094
2	150	900	2	5	Garnet	0.572	1.170
3	150	500	2	1	Garnet	0.791	1.102

**Table 4:** The three best ranking alternatives for the objectives d = 3.0 mm and min w_t_.

No.	P (MPa)	v_t_ (mm/min)	m_a_ (g/s)	h (mm)	Abrasive Material	d (mm)	w_t_ (mm)
1	250	700	8	1	Garnet	2.538	1.145
2	250	500	5	3	Garnet	2.484	1.185
3	250	500	5	3	Silicon carbide	3.004	1.414

**Table 5:** The three best ranking alternatives for the objectives min w_t_ and max MRR.

No.	P (MPa)	v_t_ (mm/min)	m_a_ (g/s)	h (mm)	Abrasive Material	w_t_ (mm)	MRR (mm^3^/min)
1	250	700	8	1	Garnet	1.145	1593.649
2	250	900	5	1	Garnet	1.116	1271.086
3	250	900	8	3	Silicon carbide	1.422	2273.834

**Table 6:** The three best ranking alternatives for the objectives d = 0.5, min w_t_, and max MRR.

No.	P (MPa)	v_t_ (mm/min)	m_a_ (g/s)	h (mm)	Abrasive Material	d (mm)	w_t_ (mm)	MRR (mm^3^/min)
1	150	900	5	1	Garnet	0.532	1.094	351.845
2	150	500	2	1	Garnet	0.791	1.101	270.990
3	150	900	2	5	Garnet	0.572	1.170	465.689

**Table 7:** The three best-ranking alternatives for the objectives d = 3.0, min w_t_, and max MRR.

No.	P (MPa)	v_t_ (mm/min)	m_a_ (g/s)	h (mm)	Abrasive Material	d (mm)	w_t_ (mm)	MRR (mm^3^/min)
1	250	700	8	1	Garnet	2.538	1.145	1593.649
2	250	900	8	3	Silicon carbide	2.382	1.423	2273.834
3	250	500	5	3	Silicon carbide	3.004	1.414	1535.497

## Data Availability

The data presented in this study are available on request from the corresponding authors.

## References

[1] Zhang T., Liu C.-T. (2022). Design of titanium alloys by additive manufacturing: A critical review. Adv. Powder Mater..

[2] Zhang L., Chen L. (2019). A Review on Biomedical Titanium Alloys: Recent Progress and Prospect. Adv. Eng. Mater..

[3] Yan Y., Lin J., Liu T., Liu B., Wang B., Qiao L., Tu J., Cao J., Qi J. (2022). Corrosion behavior of stainless steel-tungsten carbide joints brazed with AgCuX (X = In, Ti) alloys. Corros. Sci..

[4] Pervaiz S., Anwar S., Qureshi I., Ahmed N. (2019). Recent Advances in the Machining of Titanium Alloys using Minimum Quantity Lubrication (MQL) Based Techniques. Int. J. Precis. Eng. Manuf.-Green Tech..

[5] Singh H., Sharma V.S., Singh S., Dogra M. (2019). Nanofluids assisted environmental friendly lubricating strategies for the surface grinding of titanium alloy: Ti6Al4V-ELI. J. Manuf. Process..

[6] Gupta M.K., Song Q., Liu Z., Sarikaya M., Mia M., Jamil M., Singla A.K., Bansal A., Pimenov D.Y., Kuntoğlu M. (2021). Tribological performance based machinability investigations in cryogenic cooling assisted turning of α-β titanium Alloy. Tribol. Int..

[7] Elhami S., Razfar M.R. (2020). Application of nano electrolyte in the electrochemical discharge machining process. Precis. Eng..

[8] Llanto J.M., Tolouei-Rad M., Vafadar A., Aamir M. (2021). Recent progress trend on abrasive waterjet cutting of metallic materials: A review. Appl. Sci..

[9] Liu G., Zhang Y., Natsu W. (2019). Influence of electrolyte flow mode on characteristics of electrochemical machining with electrolyte suction tool. Int. J. Mach. Tools Manuf..

[10] De Bartolomeis A., Newman S.T., Jawahir I.S., Biermann D., Shokrani A. (2021). Future research directions in the machining of Inconel 718. J. Mater. Process. Technol..

[11] Natarajan Y., Murugesan P.K., Mohan M., Khan S.A., Liyakath A. (2020). Abrasive Water Jet Machining process: A state of art of review. J. Manuf. Process..

[12] Akkurt A. (2015). The effect of cutting process on surface microstructure and hardness of pure and Al 6061 aluminium alloy. Eng. Sci. Technol. Int. J..

[13] Haddad M., Zitoune R., Bougherara H., Eyma F., Castanié B. (2014). Study of trimming damages of CFRP structures in function of the machining processes and their impact on the mechanical behavior. Compos. Part B Eng..

[14] Chen F.L., Wang J., Lemma E., Siores E. (2003). Striation formation mechanisms on the jet cutting surface. J. Mater. Process. Technol..

[15] Lemma E., Deam R., Chen L. (2005). Maximum depth of cut and mechanics of erosion in AWJ oscillation cutting of ductile materials. J. Mater. Process. Technol..

[16] Junkar M., Jurisevic B., Fajdiga M., Grah M. (2006). Finite element analysis of single-particle impact in abrasive water jet machining. Int. J. Impact Eng..

[17] Zeng J., Kim T.J. (1996). An erosion model of polycrystalline ceramics in abrasive waterjet cutting. Wear.

[18] Nguyen T., Wang J. (2019). A review on the erosion mechanisms in abrasive waterjet micromachining of brittle materials. Int. J. Extrem. Manuf..

[19] Alberdi A., Rivero A., López de Lacalle L.N., Etxeberria I., Suárez A. (2010). Effect of process parameter on the kerf geometry in abrasive water jet milling. Int. J. Adv. Manuf. Technol..

[20] Rabani A., Madariaga J., Bouvier C., Axinte D. (2016). An approach for using iterative learning for controlling the jet penetration depth in abrasive waterjet milling. J. Manuf. Process..

[21] Panchal K.D., Shaikh A.H. (2022). Performance analysis and process parameters optimisation on specific cutting energy in the abrasive waterjet cutting. Int. J. Ambient. Energy.

[22] Uhlmann E., Männel C., Braun T. (2020). Efficient abrasive water jet milling for near-net-shape fabrication of difficult-to-cut materials. Int. J. Adv. Manuf. Technol..

[23] Yuan Y., Chen J., Gao H., Wang X. (2020). An investigation into the abrasive waterjet milling circular pocket on titanium alloy. Int. J. Adv. Manuf. Technol..

[24] Alberdi A., Rivero A., Lopez de Lacalle L.N. (2011). Experimental study of the slot overlapping and tool path variation effect in abrasive waterjet milling. J. Manuf. Sci. Eng..

[25] Chen F.L., Siores E., Patel K., Momber A.W. (2002). Minimising particle contamination at abrasive waterjet machined surfaces by a nozzle oscillation technique. Int. J. Mach. Tools Manuf..

[26] Rivero A., Alberdi A., Artaza T., Mendia L., Lamikiz A. (2018). Surface properties and fatigue failure analysis of alloy 718 surfaces milled by abrasive and plain waterjet. Int. J. Adv. Manuf. Technol..

[27] Fowler G., Shipway P.H., Pashby I.R. (2005). A technical note on grit embedment following abrasive water-jet milling of a titanium alloy. J. Mater. Process. Technol..

[28] Boud F., Carpenter C., Folkes J., Shipway P.H. (2010). Abrasive waterjet cutting of a titanium alloy: The influence of abrasive morphology and mechanical properties on workpiece grit embedment and cut quality. J. Mater. Process. Technol..

[29] Hashish M. (1991). Characteristics of surfaces machined with abrasive water jets. J. Eng. Mater. Tech..

[30] Bergs T., Schüler M., Dadgar M., Herrig T., Klink A. (2020). Investigation of Waterjet Phases on Material Removal Characteristics. Procedia CIRP.

[31] Stachowiak G.B., Stachowiak G.W. (2001). The effects of particle characteristics on three body abrasive wear. Wear.

[32] Fowler G., Pashby I.R., Shipway P.H. (2009). The effect of particle hardness and shape when abrasive water jet milling titanium alloy Ti6Al4V. Wear.

[33] Fowler G., Shipway P.H., Pashby I.R. (2005). Abrasive water-jet controlled depth milling of Ti6Al4V alloy—An investigation of the role of jet—Workpiece traverse speed and abrasive grit size on the characteristics of the milled material. J. Mater. Process. Technol..

[34] Perec A. (2018). Experimental research into alternative abrasive material for the abrasive water-jet cutting of titanium. Int. J. Adv. Manuf. Technol..

[35] Perec A., Pude F., Grigoryev A., Kaufeld M. (2019). A study of wear on focusing tubes exposed to corundum-based abrasives in the waterjet cutting process. Int. J. Adv. Manuf. Technol..

[36] Perec A. (2021). Research into the disintegration of abrasive materials in the abrasive water jet machining process. Materials.

[37] Palaniyappan S., Veiravan A., Kaliyamoorthy R., Kumar V. (2022). Sustainable solution to low-cost alternative abrasive from electric ceramic insulator waste for use in abrasive waterjet machining. Int. J. Adv. Manuf. Technol..

[38] Khan A.A., Haque M.M. (2007). Performance of different abrasive materials during abrasive water jet machining of glass. J. Mater. Process. Technol..

[39] Sabarinathan P., Annamalai V.E., Rajkumar K. (2020). Sustainable application of grinding wheel waste as abrasive for abrasive waterjet machining process. J. Clean. Prod..

[40] Srinivas S., Ramesh Babu N. (2012). Penetration ability of abrasive waterjets in cutting of aluminum-silicon carbide particulate metal matrix composites. Mach. Sci. Technol..

[41] Thamizhvalavan P., Arivazhagan S., Yuvaraj N., Ramesh B. (2019). Machinability of abrasive aqua jet parameters on hybrid metal matrix composite. Mater. Manuf. Process..

[42] Yu Y., Sun T., Yuan Y., Gao H., Wang X. (2020). Experimental investigation into the effect of abrasive process parameters on the cutting performance for abrasive waterjet technology: A case study. Int. J. Adv. Manuf. Technol..

[43] Balaji K., Siva Kumar M., Yuvaraj N. (2021). Multi objective taguchi-grey relational analysis and krill herd algorithm approaches to investigate the parametric optimization in abrasive water jet drilling of stainless steel. Appl. Soft Comput..

[44] Cosansu G., Cogun C. (2012). An investigation on use of colemanite powder as abrasive in abrasive waterjet cutting (AWJC). J. Mech. Sci. Technol..

[45] Zhu Y., Lu W., Zuo D., Xiao H., Cao D., Ko T.J., Wu J., Yin Y. (2019). Development of abrasive jet polishing by using amino thermosetting plastic abrasive for aluminum alloy. J. Manuf. Process..

[46] Egea A.J.S., Rodriguez A., Celentano D., Calleja A., Lopez de Lacalle L.N. (2019). Joining metrics enhancement when combining FSW and ball-burnishing in a 2050 aluminum alloy. Surf. Coat. Technol..

[47] Bolzon G., Buljak V., Maier G., Miller G. (2011). Assessment of elastic-plastic material parameters comparatively by three procedures based on indentation test and inverse analysis. Inverse Probl. Sci. Eng..

[48] Maier G., Bocciarelli M., Bolzon G., Fedele T. (2006). Inverse Analysis in Fracture Mechanics. Int. J. Fract..

[49] Bocciarelli M., Bolzon G., Maier G. (2005). Parameter identification in anisotropic elastoplasticity by indentation and imprint mapping. Mech. Mater..

[50] Younas M., Jaffery S.H.I., Khan M., Khan M.A., Ahmad R., Mubashar A., Ali L. (2019). Multi-objective optimization for sustainable turning Ti6Al4V alloy using grey relational analysis (GRA) based on analytic hierarchy process (AHP). Int. J. Adv. Manuf. Technol..

[51] Yan J., Li L. (2013). Multi-objective optimization of milling parameters- the trade-offs between energy, production rate and cutting quality. J. Clean. Prod..

[52] Hassan A.I., Chen C., Kovacevic R. (2004). On-line monitoring of depth of cut in AWJ cutting. Int. J. Mach. Tools Manuf..

[53] Ishfaq K., Mufti N.A., Ahmed N., Pervaiz S. (2019). Abrasive waterjet cutting of cladded material: Kerf taper and MRR analysis. Mater. Manuf. Process..

[54] Satyanarayana S., Babu N. (2011). Role of garnet and silicon carbide abrasives in abrasive waterjet cutting of aluminum-silicon carbide particulate metal matrix composites. Int. J. Appl. Res. Mech. Eng..

[55] Hlaváč L.M., Gembalová L., Štěpán P., Hlaváčová I.M. (2015). Improvement of abrasive water jet machining accuracy for titanium and TiNb alloy. Int. J. Adv. Manuf. Technol..

[56] Srinivasu D.S., Axinte D.A., Shipway P.H., Folkes J. (2009). Influence of kinematic operating parameters on kerf geometry in abrasive waterjet machining of silicon carbide ceramics. Int. J. Mach. Tools Manuf..

[57] Perec A. (2017). Disintegration and recycling possibility of selected abrasives for water jet cutting. Dyna.

[58] Shibin R., Anandakrishnan V., Sathish S., Sujana V.M. (2020). Investigation on the abrasive water jet machinability of AA2014 using SiC as abrasive. Mater. Today Proc..

[59] Babu M.N., Muthukrishnan N. (2018). Exploration on Kerf-angle and surface roughness in abrasive waterjet machining using response surface method. J. Inst. Eng..

[60] Karakurt I., Aydin G., Aydiner K. (2014). An investigation on the kerf width in abrasive waterjet cutting of granitic rocks. Arab. J. Geosci..

[61] Aydin G., Kaya S., Karakurt I. (2019). Effect of abrasive type on marble cutting performance of abrasive waterjet. Arab. J. Geosci..

[62] Uthayakumar M., Khan M.A., Kumaran S.T., Slota A., Zajac J. (2016). Machinability of nickel-based superalloy by abrasive water jet machining. Mater. Manuf. Process..

[63] Azmir A.M., Ahsan K.A., Rahmah A. (2009). Effect of abrasive waterjet machining Parameters on aramid fiber reinforced plastics composite. Int. J. Mater. Form..

